# Biosensing chips for cancer diagnosis and treatment: a new wave towards clinical innovation

**DOI:** 10.1186/s12935-022-02777-7

**Published:** 2022-11-15

**Authors:** Muhammad Javed Iqbal, Zeeshan Javed, Jesús Herrera-Bravo, Haleema Sadia, Faiza Anum, Shahid Raza, Arifa Tahir, Muhammad Naeem Shahwani, Javad Sharifi-Rad, Daniela Calina, William C. Cho

**Affiliations:** 1 Department of Biotechnology, Faculty of Sciences, University of Sialkot, Sialkot, Pakistan; 2 Lahore Garrison University, Main Campus, Sector C, Phase VI, DHA, Lahore, Pakistan; 3 Departamento de Ciencias Básicas, Facultad de Ciencias, Universidad Santo Tomas, Santiago, Chile; 4 Center of Molecular Biology and Pharmacogenetics, Scientific and Technological Bioresource Nucleus, Universidad de La Frontera, 4811230 Temuco, Chile; 5 Department of Biotechnology, Engineering and Management Sciences, Balochistan University of Information Technology, Quetta, Pakistan; 6 Department of Botany, Lahore College for Women University, Lahore, Pakistan; 7 Department of Environmental Sciences, Lahore College for Women University, Lahore, Pakistan; 8 Facultad de Medicina, Universidad del Azuay, Cuenca, Ecuador; 9 Department of Clinical Pharmacy, University of Medicine and Pharmacy of Craiova, 200349 Craiova, Romania; 10 Department of Clinical Oncology, Queen Elizabeth Hospital, Kowloon, Hong Kong China

**Keywords:** Biosensors, Oncogenes, Cancer detection, Diagnostics, Clinical diagnostics, Biomarkers

## Abstract

Recent technological advances in nanoscience and material designing have led to the development of point-of-care devices for biomolecule sensing and cancer diagnosis. In situ and portable sensing devices for bedside, diagnosis can effectively improve the patient’s clinical outcomes and reduce the mortality rate. Detection of exosomal RNAs by immuno-biochip with increased sensitivity and specificity to diagnose cancer has raised the understanding of the tumor microenvironment and many other technology-based biosensing devices hold great promise for clinical innovations to conquer the unbeatable fort of cancer metastasis. Electrochemical biosensors are the most sensitive category of biomolecule detection sensors with significantly low concentrations down to the atomic level. In this sense, this review addresses the recent advances in cancer detection and diagnosis by developing significant biological sensing devices that are believed to have better sensing potential than existing facilities.

## Introduction

Cancers are the second leading cause of death after cardiovascular disease and the incidence of malignancies increases with age [1, 2]. The different types of neoplasms vary significantly in incidence between different population groups, under the influence of several factors: environmental factors, eating habits and lifestyle [3, 4]. The malignant nature of various tumors is based on their ability to metastasize. This ability is influenced by the type of tissue of origin, the organ in which it is located, and the size of the tumor [5, 6]. The determination of tumor markers and serum enzymes is relevant for the diagnosis and monitoring of the evolution of metastatic tumors. Enzymes are especially important in bone and liver metastases (e.g., the clinical sensitivity of alkaline phosphatase in solitary bone metastases is 20% and in multiple bone metastases 70%). Tumor markers are substances produced directly by tumor cells or non-tumor cells under the influence of tumor cells [7].

With the global rise in both cancer incidence and mortality, there is the utmost need to get benefit from the technological advancements by combining genomic and proteomic findings with data science for better diagnosis and to limit wrong and late cancer diagnoses. Computational biology and nano-bioelectronics are believed to be key tools to develop new specific biomarkers having molecular association with specific human cancer types and stages [8, 9].

**Fig. 1:**
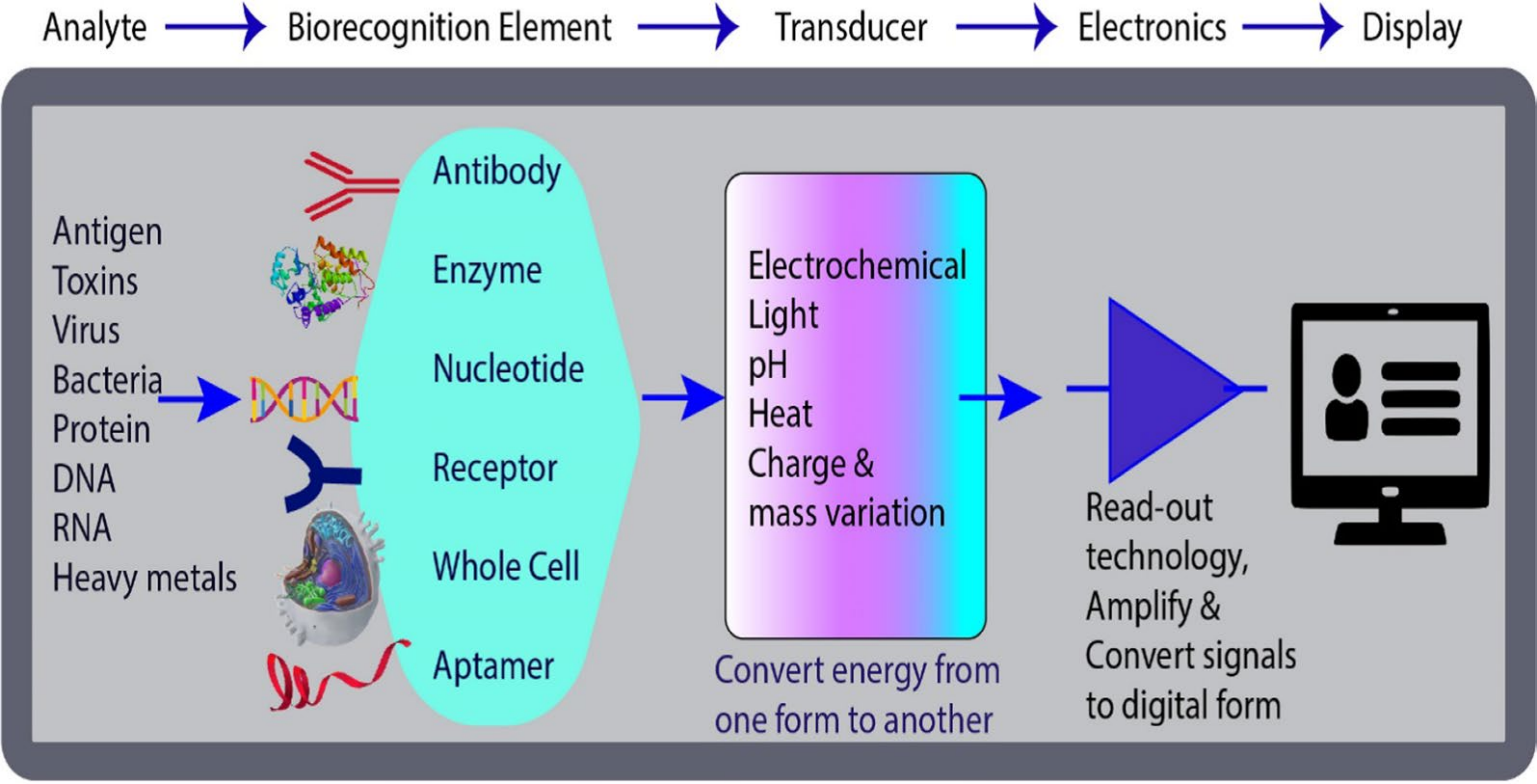
Schematic representation of a biosensor

The biosensor is a device having specific molecular interactions with biological elements for the sensitive detection of the targeted analyte. These small sensing devices can quantify biochemical reactions and generate the signal in response to the concentration of the analyte. Biosensors typically consist of;

(i) Analyte: a targeted substance that needs to detect,
(ii) Biorecognition element: a molecule that recognizes analyte,
(iii) Transducer: to transform energy into a readable signal,
(iv) Electronics: to process transduce signal by involving complex series of circuits and after amplification converts into digital form,
(v) Display: to provide a visual image, graph, or table (Fig. 1). Chip and nanotechnology lab devices bring unique physiochemical features to improve the biosensing performance of distinct point of care (PoC) devices. Indeed, the detection of the biomarkers in different body fluids, like urine, blood, and saliva, by conventional clinical methodologies depends on both their presence and concentration level, is time-taking, expensive, and is not very sensitive at low concentrations to diagnose cancer at very early stages. Clinical technologists are keen to introduce rapid and nano-sensitive detection systems by emphasizing molecular genomic and proteomic techniques to link the observed data of millions of patients (big data) with computational biology tools for more accurate and sensitive diagnosis [10, 11].

Conceiving cancer as a disease where cells present both a complex architecture and a wide range of inducing factors, including radiation, chemicals, environmental factors, bacteria, and viruses, the demand for dynamic tools to diagnose both the distinct cancer types and stages is of utmost interest since more than 220 human disorders have been linked to the carcinogenesis’ onset [10, 11]. In addition, it has been confirmed that different miRNAs have a close molecular association with both cancer onset and identification, besides being potential biomarkers for diagnosis (Table 1). For example, miR-150 has been conceived as a key contributing factor for colorectal, gastric, acute myeloid leukemia and lung cancer (LC) [12–15]. Also, quantum dot-based nano-bio sensors have proved to be significant with diverse applications and compositions to detect circulatory miRNAs as biomarkers for early-stage cancer assessment [16, 17]. Additionally, fluorescence light-up biosensors, hybrid nanoparticles and deoxyribonucleic acid (DNA) hydrogel-based biosensors are new entities in this emerging domain of PoC devices to gap the distance between portable and lab-on-chip settings for human disease diagnosis [18–20]. In this sense, this review particularly focuses on the currently used biosensing techniques for sensitive cancer diagnosis and its future perspectives on digital health care systems.

**Table 1:** Various biomarkers with diagnostic implications in different cancers

Biomarker	Diagnosis type of cancer	Methodology	References
miR-21 miR24 Let-7b Let-7e	Non-small cell lung cancer	Surface plasmon resonance	[43, 44]
miR-17-sp miR-130a-3p miR-340-5p miR-93-5p	Breast cancer	Surface plasmon resonance	[45, 46]
miR-10b	Pancreatic cancer	Surface plasmon resonance	[47–49]
miR-let-7a	Hepatocellular cancer Human cancer cells	Electrochemical biosensors	[50–52]
miR-let-7a	Multiple cancer type	Photoelectrochemical sensor	[52, 53]
miR1229	Colorectal cancer	Quantitative real-time PCR (qRT-PCR) analysis	[54–56]
miR-1246	Multiple cancer types	Enzyme-free quantum dot electrochemical biosensor	[57]
miR-150	Colorectal cancer Gastric cancer Acute myeloid leukemia Lung cancer	Graphene/carbon quantum dot base Nano-bio sensors Electrochemical Biosensors	[58, 59] [59] [16, 44, 60]
miR-21	Lung cancer	Fluorescence light- up biosensors Microfluidic paper base visual/electrochemical biosensor	[19, 61]
miR-223	Ovarian cancer	DNA hydrogel-based electrochemical biosensors MiRNA based biosensors	[62, 63]
miR-23a	Pancreatic cancer	Photo-electrochemical biosensors	[62]

## Methodology

This updated comprehensive review described the current status of the use of biosensors in the analysis of cancer to bring clinical benefits in the early stages of the disease and for early detection, by combining several parameters. For this up-to-date review, specialized databases such as PubMed/MedLine, Scopus, TRIP Database, Scopus were searched using the following MeSH terms: **“**Biomarkers”, “Biosensing Techniques”, “Diagnosis”, “Disease”, “Humans”, “Proteins”, “Neoplasms/diagnosis”. The most important data have been summarized in tables and representative figures.

## Antigen-associated biomarkers

Oncology is the main medical area that benefits from the personalized approach [6, 21, 22]. Biomarkers are the basis for accurate diagnosis of cancer and are biological features, which can be molecular, anatomical, physiological or biochemical [23–25]. These traits can be measured and evaluated objectively, becoming indicators of a normal or pathological biological process [26–29]. Depending on the role, biomarkers can be: diagnostic, risk, prognosis (favorable or aggressive), prediction of the degree of response or toxicity to treatment [30–33]. A biomarker is a measurable and quantifiable biological molecule that can be a protein, DNA, RNA, or biological component that acts as an indicator of a specific biological status [34–36]. The dosing of antigen-associated biomarkers must be targeted, depending on the anamnestic, clinical and paraclinical data, and must follow certain principles, in accordance with the currently existing guidelines [37–42]:

i. Confirmation of the result will be done through serial testing, at the same laboratory and using the same method.
ii. Post-therapeutic monitoring of the biomarker initially detected with increased values.
iii. Judicious analysis of biomarker levels according to half-life, metabolism and elimination.
iv. The use of several biomarkers to increase the sensitivity and specificity of the diagnosis.
v. Performing a correct differential diagnosis, considering that certain biomarkers also appear in ectopic tumors or may have increased values in physiological processes or in benign conditions.

A wide range of tumor cell-associated molecules has been identified in body fluids and biopsies as cancer biomarkers for diagnosing different types of cancer (Table 1) and is expected to a rise in the number of new biomarkers with the advancement in computational biology and genetics.

Biomarkers associated with the diagnosis and prognosis of rapid and early clinical cancer stages show over-expression of numerous genes and proteins, including prostate-specific antigen (PSA), Cancer antigen 125 (CA-125), Carcinoembryonic antigen (CEA) test, Cancer antigen 19-9 (CA19-9), among others, despite these antigen-associated biomarkers are less specific and more sensitive to the demand for multiple biomarkers to diagnose specific cancer types and stages [64, 65]. Importance for cancer management:

i. Screening (early detection): because tumor markers are not sensitive enough or specific enough, they are not very useful for screening in the general population. However, few of them can be used as a screening for people at high risk of developing a type of cancer-based on strong family history or specific risk factors for a particular type of cancer [66].
ii. Diagnosis: for the patients who have cancer-specific symptoms, they can help with other specific diagnostic methods to detect cancer and help differentiate from other conditions with similar symptoms [67]
iii. Monitoring the response to specific treatment: for certain cancers (eg breast, testicular, colon and rectal cancers, ovary, pancreas) serial and repeated monitoring of the appropriate markers allows the assessment of the effectiveness of the treatment, especially in the advanced stages of the disease (if the serum marker decreases, the treatment is effective; the serum marker remains high, it is necessary to adjust or change the treatment). However, the information provided by the marker values should be used with caution because there are situations that may increase or decrease the level of the marker regardless of the evolution or involution of the tumor [68].
iv. Detection of disease recurrence (recurrence): one of the most important uses of tumor markers, along with monitoring the response to treatment is the (early) detection of recurrence.

If the marker values are increased before starting the treatment, normalize during or after the end of the treatment and then start to grow after a while, it is assumed a tumor recurrence that must be confirmed by specific imaging methods (ultrasound, CT examination, MRI examination, PET-CT examination) or other methods (surgery, biopsies, tissue, liquid biopsy, etc.) [69–71]. Limitations of tumor biomarker-based detection in clinical cases derive from not discovering all the mutations and pathways involved in oncogenesis; as well as the mechanisms that allow the tumor to evade the immune system response, as data show that activation of oncogenesis pathways leads to immune system impairment [3, 72, 73]. Significant effort will be needed in the coming years to define and validate biomarkers that allow patient selection for personalized cancer management [74–77].

Tumor biomarkers detection provides useful clinical information, but also has clinical pitfalls [78–80]:

i. Tumor biomarkers may increase as a result of non-cancerous diseases
ii. The level of tumor biomarkers fluctuates over time
iii. They may not increase until the cancer is in an advanced stage, which makes the test not useful in high-risk patients for early detection of cancer or relapse.
iv. Some cancers do not produce tumor biomarkers that appear in the blood. Also, not all cancers have known tumor markers.
v. Some patients do not have high levels of tumor markers, even though the type of cancer they have usually caused tumor markers.

## Biosensors and cancer diagnosis

An ultrasensitive biosensing system has proved to be an excellent candidate for rapid, automatic, sensitive sensing and analysis of biological changes in the body or surrounding environment by using a single chip [8, 81]. Indeed, with the increased acceptance and rising trend of digital healthcare-based systems, various groups have been increasingly devoted to both developing and launching PoC biological sending devices. Among them, memristive biosensors have been reported to be able to identify PSA and other cancer-related biomarkers that will pave the way for the future of biosensing devices [82, 83]. Indeed, the conventional methods used for detecting biological biomarkers, including quantitative real-time protein chain reaction (qRT-PCR), microarray, and next-generation sequencing are much more expensive and time-taking, and, thus, nano-sensing devices are believed to be the next wave of technology for rapid and accurate exosomal micro-RNA analysis [84, 85].

Briefly, exosomes are released into the extracellular microenvironment during cell exocytosis and can be isolated from body fluids, and thus can be used as a potential biomarker for early cancer detection. Among them, tumour-associated Exosomes (TEXs) have been reported to play a significant role in tumorigenesis and are new additions to the existing pool of biomarkers for non-small cell lung cancer (NSCLC) detection [86, 87].

Specifically, the TEXs microRNAs including “miR-21” are exosomal biomarkers for rapid and accurate lung cancer (LC) diagnosis [88]. In microfluidic biochips, TEXs have a chemical affinity to the biochip surface due to the presence of ion-exchange nanomembranes. Currently, most existing clinical microdevices require RNA, DNA, or protein-extraction procedures, with a lot of data being obtained from clinical trials aiming to directly identify TEXs from body fluids by using micro-fluidic and electrochemical biosensing devices [43].

Electrochemical biosensors are based on molecular beacons. Recently, it has been reported that fluorescence detection can detect cancer at early stages, including lung, breast, and pancreatic cancers [89, 90]. Indeed, the electrochemical biosensor is a simple, efficient, ultra-sensitive, and selective method for quantitative analysis and characterising exosomal RNAs. These biosensors can easily be fabricated for clinical applications. Electrochemical DNA biosensors coupled with locked nucleic acid have the potential to identify exosomal miRNAs including miR-143, miR-146a, and miR-21, among others [90]. Moreover, immuno-biochip has also been used for in-situ identification of exosomal RNAs via cationic lipoplexes to sense RNA-specific molecular beacons (CLP-MBs) in LC samples. Also, electrostatic interaction has been used to monitor fluorescence and to analyze exosomal RNA by microscopy analysis, including total internal reflection fluorescence (TIRF) [43].

## Micro-fluidic as a point of care devices

**Fig. 2:**
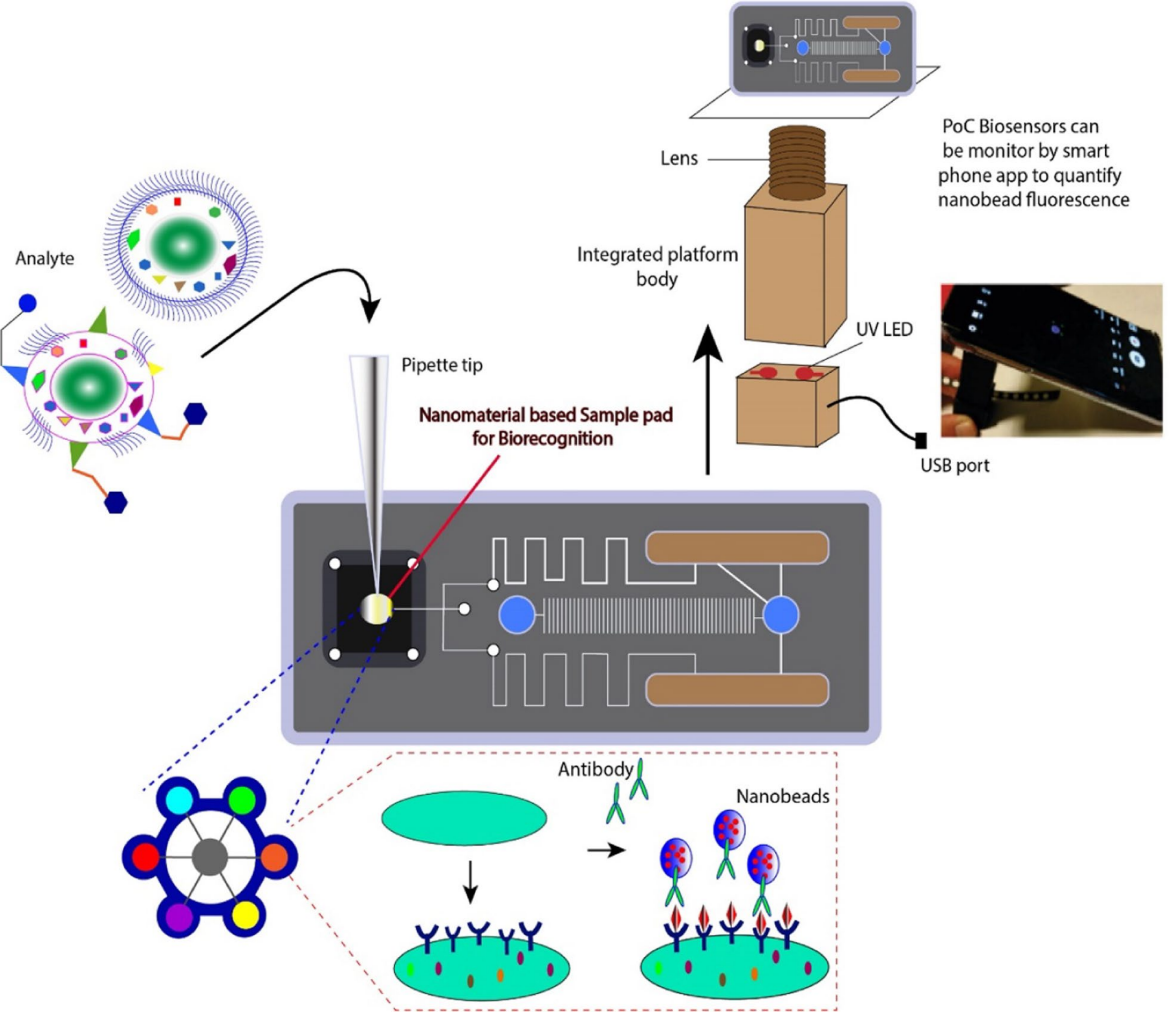
Molecular point of care devices for real-time diagnosis and quality patient care system

Microfluidic devices have attained huge attention among the clinical scientific community, appearing as a potential platform to bring revolution in medical technology. Microfluidic PoC devices are reported to have a wide range of applications and benefits as compared to the gigantic instruments (Fig. 2), as it requires analytes in the nanoliter range, reduces sample consumption, and even minimizes instrument size, and maintenance expenses. A rapid in situ diagnosis and processing of targeted biomolecule within the micro-reactor has also been stated as a significant contributing factor during surgery and is thus viewed as a key candidate for portable PoC devices [91].

Biocompatibility of sensing devices is one of the core challenging domains that demand expert handling and manufacturing skills, with four aspects revealing to be of utmost interest as they play a decisive role to design biochips for a specific function:

1. A proper selection of dynamic and biocompatible material or element to fulfil desired biological interaction.
2. The manufacturing methodology to enhance nanoscale efficiency by a bottom-up approach to increase the surface area for molecular interaction and specificity.
3. Micro-channel surfaces with increased adsorption potential for biomolecules including body fluids, as microfluidic device specificity relies on surface architecture and biochips surface properties.
4. Surface reactivity under certain conditions, including temperature, pH, solvent compatibility, electrical and magnetic field [91].

The ability to successfully isolate cancer cells is an essential step in making liquid biopsy where cancer could be detected with a simple blood sample. This would eliminate the discomfort and cost of tissue biopsies, which use needles or surgical procedures [92]. Liquid biopsy could also be useful for tracking the effectiveness of chemotherapy over time and for detecting cancer in organs difficult to reach with traditional biopsy techniques, including the brain and lungs [93, 94].

However, isolating circulating tumor cells from the blood is not an easy task because they are present in extremely small amounts. For many cancers, circulating cells are present at levels close to 1 in 1 billion blood cells [38]. Microfluidic technologies represent an alternative to traditional methods of detecting cells in fluids [38]. These devices use markers to capture target cells as they float or take advantage of target cells’ physical properties—mainly size—to separate them from other cells present in fluids [95]. Papautsky and his colleagues have developed a device that uses size to separate tumor cells from blood [96]. Using size differences to separate cell types in a liquid is much easier than affinity separation because it requires less processing of the blood. To separate cancer cells from blood, the device created by Papautsky and his colleagues uses the accumulated knowledge of the phenomena of inertial migration and shear-induced diffusion applied to blood passing through the “microchannels” formed in the device [97]. The researchers collected 5 ml samples of healthy blood, to which they had previously added 10 small lung cancer cells, and then passed the blood through their device. They were able to recover 93% of the cancer cells using the microfluidic device [97]. Previously developed microfluidic devices designed to separate tumor cells circulating in the blood had recovery rates between 50 and 80% [97]. When analyzing eight blood samples taken from patients diagnosed with small cell lung cancer, the researchers were able to separate cancer cells from six samples using the new device. In their experiments, the researchers used undiluted blood as well as blood diluted only three times, which is very little compared to other cell separation protocols used in other inertial migration-based devices [96, 97].

## Immuno-biochip for cancer diagnosis

Molecular-based detection methods have the potential to diagnose different types of cancer, but these approaches are time-taking, costly, and more labour is required to perform diagnostic tests [98–100]. To overcome these limitations, biological chips are currently used and they have a therapeutic role in cancer diagnosis as they show fast, accurate and cost-effective output [43]. The sensitivity and specificity ratios of different biochips are almost equal to that of molecular and serological assays. Immuno-biochip is a lab on a chip (LOC) based microfluidic device that is specifically designed to detect epidermal growth factor receptor 2 (EGFR2) protein of breast cancer due to antigen–antibody conjugation [101, 102]. Immobilization of nanoparticles with biochips could remarkably enhance the sensitivity of immune-biochip [101, 103, 104]. Graphene nanosheets are preferable over other nanomaterials because of their high electrical and optical conductivity. 3D porous Graphene foam (GF) synthesized by the chemical vapour deposition method and 1D titanium oxide nanofiber can transfer electrons to the biochip due to their excellent structural and functional characteristics [105–107]. The working electrode of the chip consists of Graphene foam along with carbon-doped titanium oxide extrinsic semiconductor that shows high impedance signal, Au and Ag/AgCl based reference electrodes and anti ErbB2 molecules. Antibodies attached on the surface of titanium oxide nanofiber through physical adsorption, the carbon present in the bioconjugate (GF–TiO_2_) is helpful to increase the binding affinity of antibodies on the chip surface [108, 109]. Anti ErbB2 molecules would add to the chip surface when an amide bond is formed between the antibody molecules and bioconjugate because of EDC–NHS coupling. Small pores on the graphene foam would typically be helpful in the proper handling of sample mixture during detection in a microfluidic device. Immuno-biochip has also an analyzer through which visual detection of antigen may occur. For antigenic detection electrochemical impedance spectroscopy (ESI) and differential pulse voltammetry (DPV) is used [110, 111].

## Nucleic acid-based electrochemical biosensors

The use of specific and sensitive analytical biosensing devices for early-stage cancer detection is considered the future of clinical advancements [112]. Indeed, the technical drift from current nucleic acid electrochemical devices that require polymerase chain reaction (PCR) based target-specific amplification to PoC biosensing test settings has been increasingly common [113]. Biosensors coupling with electrochemical signal transduction features are potential tools to identify specific biomolecules at both micro and nano-level concentrations. Genetic material (e.g., DNA, RNA) can recognize biomolecules via an adaptor (ligand binding) or base pairing methods and are also categorized under ideal candidate for biosensor designing [114]. In such a way, extensive research has been done on metallic nanoparticles due to their unique biocompatibility, electron transfer, and conductivity, the increased surface to volume ratio, the controlled orientation of probes, and immobilization characteristics, to improve the sensing sensitivity of nucleic acid-based biosensors. Nanoscale fabrication has proved to be a significant tool to broaden the affinity spectrum of nucleic acid biosensors for a wide range of targeted molecules, including DNA, RNA, proteins, cells, pathogens, microbes, and cell organelles [115, 116]. In addition, metal oxide and metal sulfide-base nanoparticles have also been revealed to be of practical use to introduce novel features for accurate and rapid diagnosis [117]. Indeed, based on redox potential, metallic nanoparticles can act as a tracer. In one of the experimental demonstrations, silver nanoparticles (Ag-NPs) with magnetic beads revealed a strong molecular affinity to the targeted DNA via base pairing. The nucleotide quantification was also performed based on electrochemical signals of silver (Ag), with Ag aggregates revealing to act as tags and strong electron signals transducers (electrochemical signals) due to the large size of Ag-aggregate for rapid diagnosis of cancer based upon targeted DNA [118, 119]. Also, diverse protein analysis biosensing chips were demonstrated by using aptamer-specific functionalized AgNP tags, using multiple electrodes with “thrombin” and “PDGF” specific aptamers to develop an electrochemical sensing array system and to induce Ag-NP aggregates hybridization. Silver ions voltammetry sensing was used as an indicator for target-specific biomolecule [120]. As the main findings, nanoclusters revealed a significant sensitivity for biomolecule analysis, including RNA, DNA and proteins [121].

## Bionic 3D spheroid biosensors

Different diagnostic tools have been reported to be developed in modern nanomedicine to map and investigate the aggressive behavior of different cancers including LC, which has increased continuously worldwide. In previous studies, microfluidic platforms (2D and 3D biosensors) were used for LC diagnosis as well as to study the metastatic behavior of cancer and even to investigate the crosstalk between cancer cells and the tumor microenvironment surrounding such cancer cells [122]. On the other hand, conventional drug screening processes have also been used to check the drug's efficacy and noxiousness via planer cultured cell models (2D), which are expensive and time-taking processes. However, these two-dimensional in vitro assays have been reported to fail in imitating the in vivo complexity of three-dimensional tissues, attributed to their low prediction ability in clinical trials of drug testing [123].

Nowadays, 3D-cell culture models have been increasingly used due to the improved cell behavior in the 3D environment compared to 2D. In the 3D model, cells suspended in the hydrogel are exposed to unequal components concentration present in the surrounding environment. Briefly, 3D spheroid culture is comprised of proliferating cells forming aggregates without scaffold material, where cells can interact with each other and the matrix. In an experiment, 3D LC spheroid-based biosensor was designed using LC cell lines employing interdigitated electrodes to assess drug efficacy. The results indicated higher drug resistance in the spheroid model as compared to the planer cell model. The anticarcinogen inhibition ability was assessed on A549, H1299, and H460 3D LC spheroid models by electric impedance sensing. As the main findings, the 3D spheroids model proved to be a promising prediction model in personalized medicine screening [124]. Furthermore, the discovery and development of anticancer drugs are based on 3D spheroid models screening. For example, novel inhibitors (MI-192) involved in targeting the migratory and invasive potential of cancer cells have been reported to be detected through the 3D format, and are even considered a therapeutic strategy for various cancers, i.e. gliomas. In short, the 3D spheroid assays, due to their three-dimensional tumor architecture, are considered the best possible approach for both screening and detecting potential anticancer drugs [125].

## Label-free biosensors

As a whole, the biochemical assays and labelling processes used in conventional cancer screening methods are expensive [126]. Label-free microwave biosensors have been used to identify cancer cells; briefly, such biosensors have a coplanar waveguide transmission line along with a detection window to observe the dielectric characterization of various cancer cells, including human hepatoma (HepG2), human endometrial adenocarcinoma (HEC-1-A), and human LC (A549) cell lines [127]. Biosensor hinders the microwave parasitic effects and determines the dielectric properties of cancer cells so that they are reported to be used in the in-vitro diagnosis of various cancers [46]. In addition to this, antibody-modified graphene field-effect transistor (GFET)-based label-free biosensors have been used to target cancer cells through non-covalent modifications, where GFET modified with carcinoembryonic antigen (Anti-CEA) monitors the reaction between CEA and anti-CEA proteins. In such a process, the high affinity and sensitivity of anti-CEA-GEFT reveal that graphene biosensors are a promising tool for both medical diagnostics and clinical applications [128].

On the other side, metallic nanoparticle-based biosensing devices also appear to be a good alternative to enzyme-labelled biosensors, as some specific metals can catalyze the chemical reaction and amplify the electrochemical signal [129]. Indeed, enzyme label devices rely on selective active sites that may limit the sensing potential of biomedical devices, generally appearing as a sandwich, with a structural composition containing metal nanoparticles conjugated with a reporter and an electro-immobilized probe [130]. Similarly, nanoparticle-based biosensors present a strong amplified electrochemical signal transduction potential for targeted biomolecules, making them ideal candidates for cancer diagnosis [131]. Thus, metallic nano-clusters are gaining a lot of attention in the scientific community due to their biochemical reaction amplifying potential. For instance, miRNA-based biosensors have been developed using metallic nano-clusters (e.g., Ag-NCs and Cu-NCs), with data obtained so far revealing that such nano-sensing devices are keys for an effective and specific quantification of targeted miRNA expression [131, 132].

In addition, the miR-Let-7a determination at femtomolar concentration has been achieved by using a novel propolis-based synthesis of silver nanoparticles and carbon paste electrochemical sensor from serum samples of hepatocellular and liver cancer cells [133]. Recently, innovative photoactive materials have also been used in a few experiments to determine the miRNAs at a 10^–17^ scale. Thus, the assessment of the Let-7a expression through using photoelectrochemical sensors composed of Graphdyine-based Au-NP-coupled alkaline phosphatase electrodes has opened a new venue for hybrid materials design for an effective and femtometer level determination of cancer biomarkers [134]. Also, nanotechnology-based photoelectron devices have been used in clinical trials to detect the Let-7a, relying on the photoelectrochemical response variation by using Bi_2_S_3_ nanoflower surface as signal amplification and immobilizing agents with enhanced surface area [135]. Therefore, in the coming years, it is believed that with a greater number of modified photo-electrochemical biosensor devices it will be possible to markedly improve the effectiveness and sensitivity of targeted signal amplification and diagnosis.

## Quantum dots biosensors

**Fig. 3:**
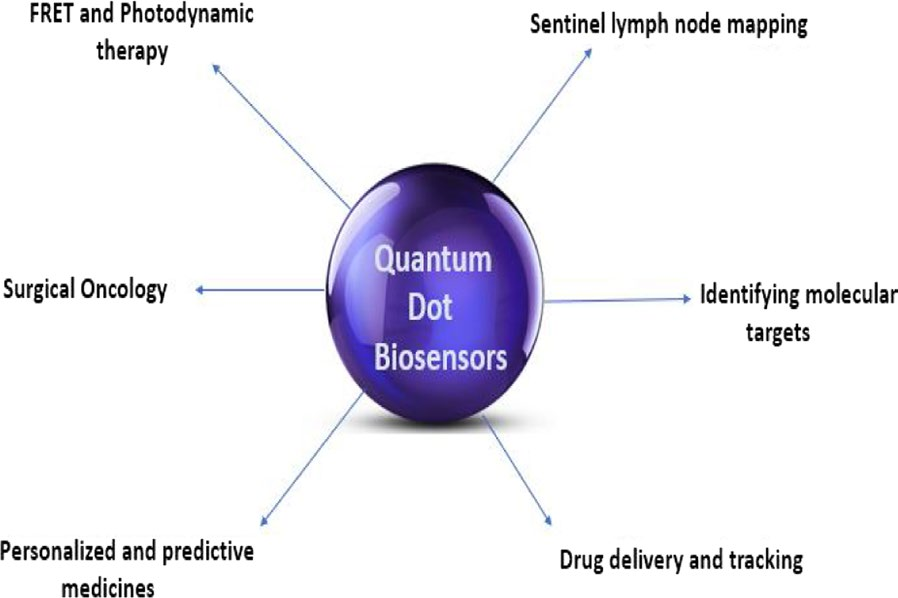
Applications of quantum dots (QDs)

Quantum dots (QD) are nanosized luminescent semiconductor crystals that have been used as promising diagnostic and therapeutic systems, due to their multiple optical properties, i.e. high brightness, concurrent signals detection, and long-term stability. In addition, QD has revealed great abilities for addressing molecular targets, tracking drugs, mapping lymph node, and fluorescence resonance energy transfer (FRET) as seen in Fig. 3. Indeed, the optical properties of QDs enable them to be ideal donors for FRET and other photodynamic therapy studies [136].

QD biosensors are being considered promising tools for the early-stage detection of various cancers [16]. QD biosensors have shown to be highly effective for cancer detection and diagnosis as well as for other clinical purposes [16, 85]. For example, magnetic fluorescent biosensors based on ferrosoferric oxide (Fe_3_O_4)_, graphene QD (GQDs), and molybdenum disulfide (MoS_2_) have been reported to be used for the detection and separation of circulating tumor cells [137]. The GQDs used as a probe have been reported to have stable photoluminescence, great biocompatibility, low cytotoxicity, and resistivity to photobleaching, meaning that they are safe in vitro and in vivo and can be used for biomedical imaging [137]. In addition, functionalized indium phosphide and zinc sulfide QD (InP/ZnP QDs) have also been shown to allow specific in vitro targeting of pancreatic cancer cell lines. Functionalized InP/ZnS QDs bioconjugates are prepared by treating QD surfaces with mercaptosuccinic acid followed by bioconjugation with monoclonal antibodies specific to pancreatic cancer, i.e. anti-claudin 4 and anti-prostate stem cell antigen (anti-PSCA) [138]. More recently, an achievement was stated to concern its ability to identify 2 targeted miRNAs at a time by using enzyme-free QD electrochemical biosensors [57].

### For cancer biomarkers detection

Multicolor QD has been reported to be a useful agent in medical imaging, being able to detect tens to several hundreds of cancer biomarkers in different blood assays [139]. Genomic and expression databases of various cancers have been increasingly developed by high throughput biomarkers screening. Specifically, QD biosensors have the potential to expand the analysis from in vitro to cellular and whole-body multiplexed cancer biomarker imaging [140].

**Fig. 4:**
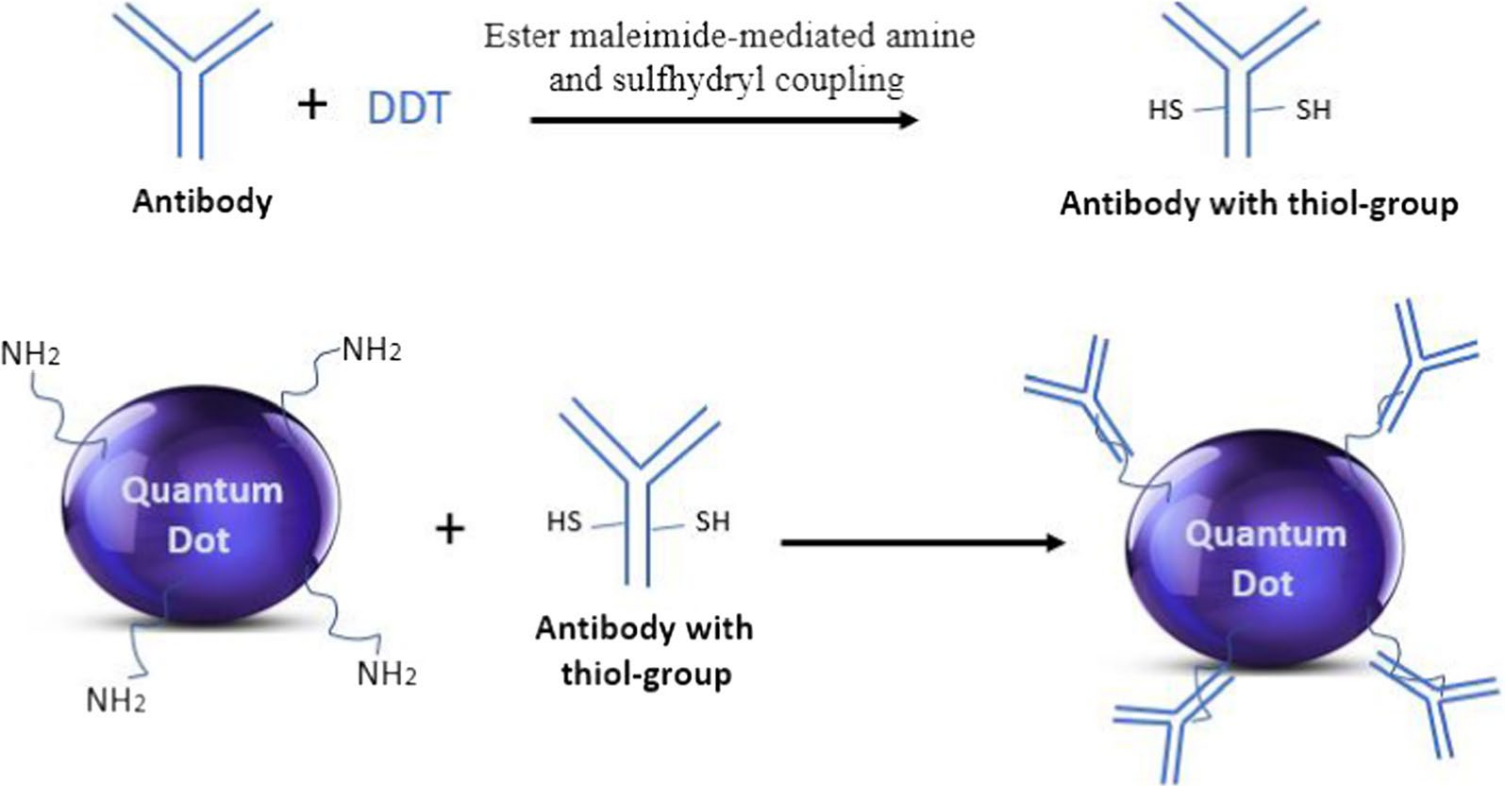
Formation of quantum dots (QDs) bioconjugates by introducing thiol groups on antibodies, thereby combing them with QDs

Multicolor QD has also been used for detecting tumor biomarkers present in breast cancer cells using fluorescent microscopy and cell spectroscopy. In multicolor QD biosensors preparation, the antibodies are first treated with dithiothreitol (DDT) to make them reactive by introducing thiol groups in the hinge region of antibodies (Fig. 4). Then, multicolor QD are covalently linked to reactive antibodies through ester maleimide-mediated amine and sulfhydryl coupling. To date, three QD-linked tumor biomarkers (i.e. ER, PR, and HER2) were reported to be detected and measured in MCF-7, BT-474, and MDA-MB-231 cell lines [141]. Furthermore, cancer marker type IV collagenase is reported to be detected by a QD-based FRET biosensor. Indeed, the cancer marker type IV collagenase was detected when fluorescence was generated due to the interaction of cancer marker, luminescent QD and small-sized FRET-based gold nanoparticles [142].

### Biocompatibility of quantum dots

The biocompatibility and specific targeting of QD in the body are reported to be achieved through its surface modification and conjugation with molecules, like antibodies, small molecules, or peptides. Bioconjugation is mostly accomplished in biology laboratories and skilled chemists are not needed for bioconjugation [142]. QDs biocompatibility is also reported to be dependent on their elemental composition, size, and surface properties, which strongly influence their toxicity mechanism. For example, Cadmium (Cd)-based QD have proved to be toxic due to the cytotoxic potential of the Cd element, which is released from the QD upon administration, thus, their use is restricted in human patients for cancer diagnosis. Indeed, Cd-based QD is being progressively replaced by copper and silver decorated carbon QD for their low in vitro and in vivo toxicity [142].

## Microreactors in cancer detection

Microreactor technology is an interdisciplinary field of science and engineering, with a vital role in biotechnology, medicine, the pharmaceutical industry, as well as for clinical and environmental diagnostics due to its increasing applications in different areas. Cell sorting, lysis, and analysis experiments have been employed on a single microreactor array chip, thus, making possible the lab on a chip platform. Moreover, clinical diagnostics-related devices are also stepping forward to carry out various biochemical analyses, such as PCR amplification, cell lysis, separation, and detection [143]. Briefly, the microreactor array system is composed of a microreactors’ array and a sealing membrane, which have two surfaces (first and second) facing opposite each other. The sealing membrane is constructed in such a way that it movably covers and seals an array of microreactors. In a microreactor array system, a reagent gap is also present, which provides a fluid path to pass through the microreactor array and the second surface of the sealing membrane. An injector and an applicator are also present for bringing reagent in a reagent gap and for directing a fluid against the first surface of the sealing membrane, respectively. Moreover, detectors are also present in a microreactor array system [143].

For example, microreactors have been reported to serve as a potential noninvasive detection system for LC. In a clinical experiment, the exhaled breath of healthy and LC patients was used by silicon microreactors coated with ATM [2‐(aminooxy)‐N, N,N‐trimethylethanammonium] to identify various volatile organic compounds (VOCs) in breath [144]. The VOCs (namely, 2‐butanone, 3‐hydroxy‐2‐butanone, 2‐hydroxyacetaldehye, and 4‐HHE) reported in the breath of LC patients were present at higher concentrations when compared to that of healthy individuals, and even in patients of benign pulmonary nodules. In addition, 2-butanone and 4-HEE concentrations have been shown useful to identify stage 1 LC and squamous cell carcinoma, respectively [145, 146].

Due to stromal contamination and genetic heterogenicity in cancer, the use of conventional DNA sequencing is limited for testing tumor biopsies. In this way, microreactor-based pyrosequencing has been reported to be able to detect cancer-based genetic variation through a parallel sampling of different DNA fragments, ultimately promoting a precise molecular diagnosis of various cancer specimens. For instance, picotiter plate pyrosequencing has been used to detect rare oncogene mutations with low tumor content in diverse samples, which are unable to be detected by the Sanger sequencing method. Thus, picotiter plate pyrosequencing enables the monitoring of the evolution of tumor subdivisions by investigating the molecular composition of samples, thereby, reducing the requirement of tumor cell enrichment methods [147]. This aspect indicates that parallel picotiter plate pyrosequencing allows for obtaining clinically accurate mutations and variations in oncogenes that are often difficult to be examined by Sanger sequencing. In this sense, for stromal admixed cancer specimens, this sequencing method helps in achieving a greater sensitivity in molecular diagnosis, thereby, facilitating the selection of specific cancer therapies [148].

In addition to this, detecting and identifying mutations in large genes is challenging in molecular diagnostics. Previously reported findings have revealed that Sangers sequencing is used to screen all BRCA1 and BRCA2 gene coding regions. Currently, massively parallel sequencing (MPS) encompassing multiplex PCR and pyrosequencing, has also been reported as useful for BRCA1/2 genes amplification and screening [149]. On the other hand, air bubbles are frequently formed in microreactors during PCR thermocycling, and it leads to the failure of microreactors-linked PCR. Indeed, bubble formation in microreactor chips composed of polydimethylsiloxane (PDMS) bonded with glass is reported to be strongly associated with the inbuilt microfeatures present in microreactors, and chip bonding interface close to the inner corners of microreactor array chips. Moreover, the PDMS gas permeability and PCR sample’s wetting properties have been reported to be the cause of air bubbles forming in microreactor chips. In such a way, to overcome the PCR failure due to bubble formation, bubble-free PCR has been progressively established in PDMS microreactors array chips using the bonding interface cladding technique. Briefly, this technique has proved to be the most reliable and suitable for PDMS microreactor array chips replicated from deep reactive ion etching (DRIE) silicon die [150].

Lastly, but also worth noting is that to properly address cancer’s pathogenesis, cell signaling pathways’ examination is of utmost importance [150]. The routinely used methods to study the pathogenesis of cancer are laborious and time-taking, and so, to overcome these constraints, gel-free cell culture followed by a subsequent immunoassay has been integrated and increasingly used in paper-based microreactor array chips [151]. The main benefits of this technique are its practicability, depicted by cell culture stimulation following the application of various conditions, including interleukin 6 (IL-6) stimulation, starvation, and hypoxia, and its ability to quantify the activated kinases [152]. Consecutive rapid screening of cellular signaling cascades following HGF and doxorubicin stimulation allows screening of both kinases and transcription factors. Thus, a paper-based microreactor array chip has proved to be a useful and less time-taking technique, as it carries out all tedious operations by just using a single paper substrate [152].

## Therapeutic perspectives on biosensors and biomarkers in cancer management

A multitude of tests has been developed, including state-of-the-art sequencing tests, to identify biomarkers that have the potential to predict the therapeutic response to various target therapies [153]. Identifying biomarkers is only a first step in the battle against cancer. Tumor biomarkers can be measured before starting treatment to determine the appropriate therapy and certain biomarkers in tumor tissue are the target of specific targeted therapies [67, 154]. If the biomarker is common enough in a specific location of cancer and is associated with a targeted therapy approved by the Food and Drug Administration (FDA) or the European Medicines Agency (EMEA), the choice of a therapeutic option appropriate to the characteristics of the tumor, in other words, therapy personalized, is the natural consequence [94].

Examples of tumor tissue markers commonly used in oncological pharmacotherapeutic management:

- Estrogen receptors and progesterone receptors (breast cancer), which are used to determine if hormone therapy and certain targeted therapies are appropriate [155].
- Analysis of the EGFR (non-small cell lung cancer) genetic mutation to determine treatment and estimate a prognosis [156].
- Programmed death- ligand 1 (PD-L1) and mutational tumor load (TMI) to assess whether treatment with a specific type of targeted inhibitor therapy is appropriate [157].

The last years are characterized by a special interest in personalized therapy, a concept developed and currently known as precision medicine [158, 159]. Targeted therapy stops the action of molecules that are the key to the growth of cancer cells. Thus, this therapy affects the cancer cells more than the normal ones. The specific action of targeted therapy differs from traditional chemotherapy, which affects all fast-growing cells [160]. There are two main types of targeted therapy. The first type is small molecule drugs—which are small enough to enter cells. The second type is monoclonal antibodies, which are too large to enter cells. In contrast, monoclonal antibodies affect targets outside cells or targets on the surface of cells [161]. Available target therapies are hormone therapies, signal transduction inhibitors, gene expression modulators, apoptosis inducers, angiogenesis inhibitors, immunotherapies, and monoclonal antibodies that provide toxic molecules [161]. Sometimes, the tumors can express a multitude of genetic aberrations [162]. Mutations or variants of a single gene may have different consequences, and the response to target therapies may differ depending on the context [158].

The development of the monoclonal antibody Trastuzumab and its use in breast cancer expressing epidermal growth factor 2 (HER 2) has greatly improved the prognosis in HER 2-positive breast cancers, being a prime and important example of personalized biomarker-based therapy [163]. The objective response rate of first-line Trastuzumab therapy in patients with or without HER 2 gene amplification ranged from 34 to 37%, illustrating the efficacy of target therapies in tumors illustrating molecular aberrations [164]. At present, a multitude of target therapies has been approved in various therapeutic lines for cancer-based on companion diagnostic tests. The results of these tests allow the choice of target therapies specific to each patient (e.g., quantification of BRAF V600 mutations for anti-RAF and anti-MEK target therapies, or BCR-ABL fusion genes for Imatinib treatment in chronic myeloid leukemia or lymphoblastic leukemia acute) [165].

In the context of personalized therapy, biomarkers have a prognostic role but are also predictive in assessing the therapeutic response, allowing the selection of patients most likely to respond to a specific treatment, integrating both sensitivity and resistance to various target therapies (e.g., BRAF V600 mutated malignant melanoma is highly sensitive to Vemurafenib therapy, while the “wild” variant is resistance to this therapy). They can also provide information on susceptibility to toxicity under the conditions of targeted therapy [4, 166]. The implementation of personalized cancer therapy starts from the ability to identify predictive molecular biomarkers for prognosis, sensitivity or resistance to a drug agent or combination therapy, as well as the assessment of their associated toxicity [167, 168]. Current efforts are aimed at identifying predictive biomarkers for therapeutic benefit. The clinical dilemma remains in the administration of target regimens for each of the molecular alterations of each tumor [169].

## Conclusion

Technology has paved the way toward a better understanding of human biological complexities including cancer and other life-threatening diseases for better diagnosis and treatment. The development of smart biosensing devices for clinical applications is the growing domain of research nowadays to recognize cancer cells and their biomarkers. Briefly, biosensors interact with the biological entity which may be DNA, RNA, and protein, and in response produce a signal that is further recognized by a detector. For clinical applications, biosensors are primarily designed to detect cancer biomarkers and to determine drug effectiveness at specific sites, so they have the potential to provide more accurate detection, monitoring, and reliable imaging of various cancer cells. Electrochemical biosensors along with many other multi-functional sensing devices including optical and quantum dot biosensors, and microfluidic nanostructures as point-of-care devices are the most sensitive sensing devices currently available and are in the clinical investigation to bring revolution in the clinical applications. Composed of electrodes with a variety of materials, including gold, silver, graphene, graphene oxide, carbon nanotubes and hybrid nanomaterial. These devices are reported to play a significant role in specific biomolecules detection, despite requiring more technological impact to diversify their sensing sensitivity and clinical applications, among them for cancer diagnosis and early intervention. Thus, in the field of oncology, a molecular understanding and proper identification of new assays such as extracellular exosomes, liquid biopsies, circulatory tumor cells and body fluids from cancer patients using current technological trends would be extremely useful, being the next wave to detect and diagnose early-stage cancer.

Several clinical challenges remain for optimal personalized cancer therapy. On the one hand, there are too few approved target therapies related to specific biomarkers. On the other hand, the spectrum of targets studied in preclinical trials is limited. Under these conditions, most gene aberrations present in tumors do not have targeted therapeutic options. Increasing the use of molecular testing is the premise of identifying predictive markers for sensitivity or resistance to various pharmacological agents and facilitating enrollment in biomarker-based clinical trials. The situation is extremely heterogeneous, encountering either unitary genomic alterations in multiple tumor types or multiple genomic alterations within the same tumor. To date, very few molecular alterations have been correlated with the clinical benefit secondary to target therapies. These benefits have led regulators to approve target therapies for several cancers that express specific molecular alterations. With the development of sequencing techniques that allow the identification of multiple genomic alterations, the need to quantify and prioritize genetic alterations that express sensitivity to a particular agent is more than stringent, especially in the case of tumors that express multiple genomic alterations with implications multiple therapeutic.

## Data Availability

Not Applicable.
